# Computational modeling and simulation in oncology

**DOI:** 10.1002/ctm2.70456

**Published:** 2025-09-05

**Authors:** Christian Baumgartner

**Affiliations:** ^1^ Department of Computer Science and Biomedical Engineering, Institute of Health Care Engineering with European Testing Center of Medical Devices Graz University of Technology Graz Austria

**Keywords:** cancer, computational modeling, digital twins, in silico trials, multiscale modeling, oncology, precision medicine

## Abstract

Computational modeling and simulation are playing an increasingly important role in oncology, bridging biological research, data science and clinical practice to better understand cancer complexity and inform therapeutic development. This special issue presents recent advances in multiscale modeling, artificial intelligence‐driven systems, digital twins, and in silico trials, illustrating the evolving potential of computational tools to support innovation from bench to bedside. Together, these contributions outline a future in which precision medicine, adaptive therapies and personalized diagnostics are guided by integrative and predictive modeling approaches.

Computational modeling and simulation are increasingly integral to modern oncology, providing essential bridges between biological understanding, data science and clinical application. By addressing cancer's inherent complexity across molecular, cellular and systemic levels, computational approaches are offering novel insights and contributing to the development of more effective diagnostic and therapeutic strategies. In this special Topic Issue, we present a curated collection of studies highlighting recent advances in multiscale modeling, artificial intelligence (AI)‐driven systems, digital twins and in silico clinical trials. Together, these contributions reflect both the current capabilities and the future potential of computational oncology to support more personalized and adaptive interventions.

## 1 COMPUTATIONAL ONCOLOGY: INSIGHTS AND PROGRESS

Cancer remains a profoundly dynamic and heterogeneous disease, shaped by nonlinear processes across genetic, cellular, tissue and systemic scales. Computational modeling has become an essential tool in capturing and interpreting these multiscale phenomena. While advancing fundamental biological understanding, computational methods are also proving increasingly valuable in therapeutic development and optimization.

Mechanistic models, grounded in biological principles, offer predictive frameworks to simulate disease processes and therapeutic responses. For instance, Powathil et al. present a hybrid multiscale model that integrates cell‐cycle dynamics with microenvironmental factors to explore therapeutic resistance mechanisms.^1^ Data‐driven approaches, such as those demonstrated by Yuan et al. using multitask learning for drug sensitivity prediction,^2^ complement mechanistic strategies by uncovering hidden patterns in complex datasets.

Multi‐scale modeling further strengthens this integration, bridging molecular mechanisms with tissue‐level behaviours. Contributions such as those by Anderson et al.^3^ and Rieger et al.^4^ underscore the potential of integrative modeling frameworks to provide more comprehensive perspectives on tumour evolution and therapeutic response.

## 2 EXPANDING THE COMPUTATIONAL TOOLKIT

A range of computational frameworks continues to be developed and refined to address the complexity of cancer biology. Agent‐based models, reviewed by Metzcar et al.,^5^ offer detailed representations of cell‐cell interactions and heterogeneity, while continuum models, as used by Frieboes et al.,^6^ provide macroscopic views of tumour dynamics.

Hybrid modeling strategies, such as those by Rejniak et al.,^7^ combine discrete and continuous approaches to capture mechanical and biological interactions more accurately. In parallel, network‐based models are increasingly valuable in mapping intracellular signalling pathways and predicting therapeutic outcomes, as highlighted by Saez‐Rodriguez et al.^8^

Each of these modeling paradigms offers specific strengths. Together, they contribute to a growing and complementary set of tools that enable deeper investigation of oncological phenomena.

## 3 TOWARDS FUNCTIONAL DIGITAL TWINS AND PRECISION MEDICINE

Building on these advances, the development of functional digital twins represents an important emerging direction. Digital twins, designed as computational counterparts to living systems, aim to provide individualized simulations that may support diagnosis, treatment planning, and monitoring.

Initial efforts, such as the digital cell twin for lung adenocarcinoma electrophysiology described by Baumgartner,^9^ demonstrate the feasibility of modeling complex cellular behaviours at high resolution. Integrative approaches incorporating high‐throughput data, exemplified by Gawel et al.^10^ and platforms such as CompuCell3D,^11^ are critical to enhancing the predictive power and translational relevance of these models.

Clinical AI frameworks, such as caiSC,^12^ offer another promising avenue by integrating multimodal single‐cell data and clinical histories to inform patient‐specific decisions. Although challenges related to data sparsity, model interpretability, and validation persist, the convergence of AI with digital twin methodologies could significantly advance precision oncology.

## 4 SUPPORTING THERAPEUTIC DEVELOPMENT AND CLINICAL DECISION‐MAKING

Computational models are increasingly influencing therapeutic development and clinical trial design. Virtual patient cohorts, as described by Sinisi et al.,^13^ provide opportunities for in silico trials that complement traditional studies, enhancing scalability and reducing ethical and logistical constraints.

Moreover, evolutionary and ecological modeling approaches, discussed by Mathur et al.,^14^ are informing adaptive therapy strategies that aim to preemptively address resistance mechanisms. Such adaptive frameworks represent a shift towards more dynamic, patient‐tailored therapeutic regimens.

## 5 TOWARDS AN INTEGRATED COMPUTATIONAL ONCOLOGY ECOSYSTEM

The studies featured in this issue illustrate the growing potential of computational modeling to inform biological discovery, therapeutic innovation, and clinical practice. However, realizing the full impact of computational oncology will require continued progress in areas such as model standardization, reproducibility, clinical validation, and data integration.

As the field advances, a more integrated ecosystem that combines mechanistic understanding, data‐driven modeling, and translational application will be critical. We hope that the work presented here not only reflects the dynamic current landscape but also encourages ongoing interdisciplinary collaboration and innovation in the years ahead.

We sincerely thank all the authors and reviewers who contributed to this special issue, ***Computational Modeling and Simulation in Oncology***. Their efforts highlight the significant strides being made towards a future where computational tools play a central role in oncology research and clinical care.

**Figure:**
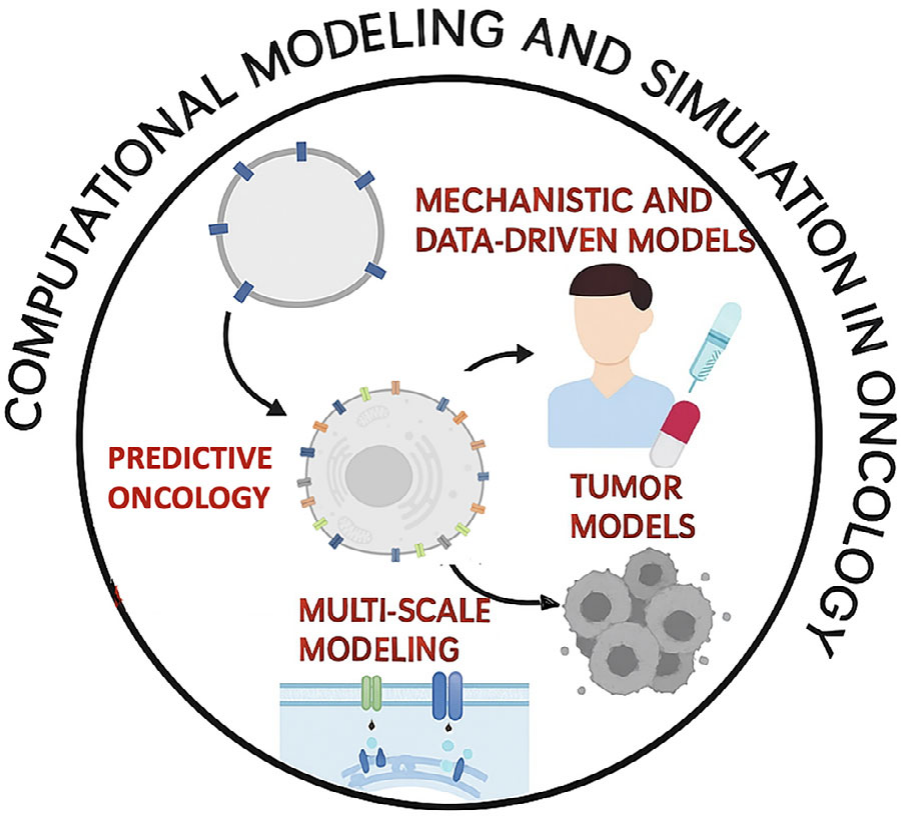
**FIGURE 1**. Logo of the Topic Issue.

## AUTHOR CONTRIBUTIONS

C.B conceptualized, wrote, and edited the manuscript.

## CONFLICT OF INTEREST STATEMENT

The author declares that he has no conflicts of interest.

## ETHICS STATEMENT

Not applicable.
